# The effect of breastfeeding on the risk of asthma in high-risk children: a case-control study in Shanghai, China

**DOI:** 10.1186/s12884-018-1936-5

**Published:** 2018-08-23

**Authors:** Xiaona Huo, Shuyuan Chu, Li Hua, Yixiao Bao, Li Du, Jian Xu, Jun Zhang

**Affiliations:** 1 MOE-Shanghai Key Laboratory of Children’s Environmental Health, Xinhua Hospital, Shanghai Jiao Tong University School of Medicine, 1665 Kong Jiang Road, Shanghai, 200092 China; 2 Laboratory of Respiratory Disease, Affiliated Hospital of Guilin Medical University, Guangxi, China; 3 Department of Pediatrics, Xinhua Hospital, Shanghai Jiao Tong University School of Medicine, Shanghai, China; 4 Department of Pediatrics, Shanghai Children’s Medical Center, Shanghai Jiao Tong University School of Medicine, Shanghai, China; 5 Shanghai Municipal Maternal and Child Health Center, Shanghai, China; 6 Guilin Medical University School of Public Health, Guangxi, China

**Keywords:** Antibiotic, Breastfeeding, Childhood asthma, Pregnancy

## Abstract

**Background:**

Increasing evidence shows that antibiotic use in pregnancy may increase the risk of childhood asthma but epidemiologic studies are still limited and findings are inconsistent. Meanwhile, exclusive and prolonged breastfeeding may prevent children from allergic diseases. We aimed to assess the association between prenatal antibiotic use and the risk of childhood asthma, and explore whether breastfeeding modifies the risk.

**Methods:**

We conducted a case-control study in Shanghai, China, from June 2015 to January 2016. A total of 634 asthma cases and 864 controls aged 3–12 years were included. Multiple logistic regressions were used to estimate crude and adjusted odds ratios (aOR).

**Results:**

The prevalence of antibiotic use in pregnancy in the cases and controls was 7.1 and 3.5%, respectively. A significant association between prenatal antibiotic use and childhood asthma was observed (aOR: 1.7, 95% CI: 1.0–2.9), particularly in boys (aOR: 2.2, 95% CI: 1.1–4.4) and children with family history of allergic disorders (aOR: 3.1, 95% CI: 1.2–8.4). However, this association existed only in children who were not breastfed exclusively in the first six months of life (aOR 2.6, 95% CI 1.3–5.1) but not in children who were exclusively breastfed (aOR 0.9, 95% CI 0.4–2.1). Likewise, exclusive breastfeeding also decreased the association between antibiotic use in pregnancy and asthma in boys and in children with family histories of allergic diseases.

**Conclusions:**

Antibiotic use in pregnancy was a risk factor for childhood asthma. However, this risk may be attenuated by exclusive breastfeeding in the first six months of life, especially among high-risk children.

**Electronic supplementary material:**

The online version of this article (10.1186/s12884-018-1936-5) contains supplementary material, which is available to authorized users.

## Background

The prevalence of childhood asthma has been increasing over the past 30 years [1, 2]. A number of factors, including genetic and environmental, have been implied to play a role in the pathophysiology of childhood asthma. However, no single factor can explain the substantial increase well [3]. Limited evidence suggests that maternal use of antibiotics in pregnancy may increase the risk of asthma in childhood [4–8]. It was postulated that the composition of pathogenic and beneficial microbiota of newborns and, consequently, the development of infant immune system were thought to be an underlying mechanism [9–11]. But this association and the hypothesis need to be confirmed in more studies.

Several other factors, such as male gender and family history of allergic disorders, have long been found to be risk factors for childhood asthma [12]. While genetic susceptibility may explain why the family history of allergic disorders is a risk factor, the environment shared by the family members may also be a contributor [13, 14]. The sex-based differences in childhood asthma has been hypothesized as due to sex difference in intrauterine gonadal steroid production and disadvantage of male in response to some in utero stress factors than female [15, 16].

Interestingly, several studies [13] suggest that prolonged and exclusive breastfeeding protects against childhood allergic disorders including asthma. The protective effect has been postulated as a result of modulation of the gut microbiota by breastfeeding, which, in turn, promotes the programming of the infant immune system [14]. The modulation effect of breastfeeding on childhood allergic disorders may also be correlated with gender and family history [17–19].

The purpose of the present study was to investigate the association between antibiotic use in pregnancy and the risk of childhood asthma, and the possible role of breastfeeding in modulating the risks using data from a case-control study on childhood asthma.

## Methods

### Study design and study population

**Fig. 1:**
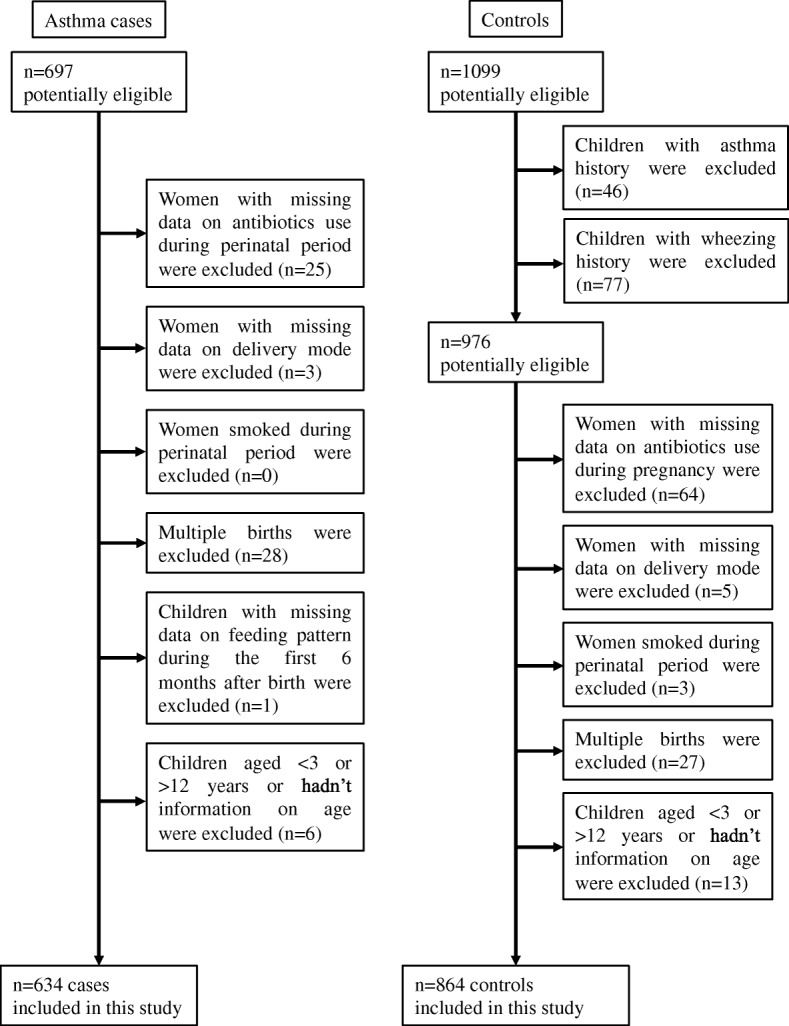
Study population selection

This hospital-based case-control study was conducted in Xinhua Hospital, Shanghai, China from June 2015 to January 2016. Children at the age of 3 to 12 years were potentially eligible. Childhood asthma was diagnosed by pediatricians according to the definition of the Global Initiative for Asthma guidelines [https://ginasthma.org/]. Controls were non-asthma patients at the similar age from outpatient general pediatrics and pediatric surgery clinics. 697 cases of childhood asthma and 1099 potential controls were recruited. Mother-child pairs were excluded if they had missing information on maternal antibiotic use in pregnancy, delivery mode, feeding patterns within the first six months of life, child age and multiple births. Only three mothers smoked in pregnancy and, therefore, were excluded. Children with a history of wheezing were also excluded from the controls, leaving a total of 634 asthma cases and 864 non-asthma controls for analysis (Fig. 1). This study was approved by the Committee of Research Ethics at the Xinhua Hospital. All parents provided a written informed consent.

A wide range of information was collected by a face-to-face interview from the parents of the cases and controls, including antibiotic use in pregnancy (excluding antibiotic use during labor and delivery), feeding patterns within the first six months of life and baseline characteristic. Unfortunately, no information was collected on reasons for antibiotic use, types of antibiotics or dosage. Breastfeeding practice in the first 6 months postpartum was classified as exclusive breastfeeding, mixed feeding and bottle feeding. Exclusive breastfeeding was defined as infants only receiving breast milk without solid and liquid food (except water).

### Statistical analysis

We first compared the maternal and infant characteristics between cases and controls by Cochran-Mantel-Haenszel Chi-square and Student’s t test. We then examined the association between antibiotic exposure in pregnancy and childhood asthma. The modifiable effect of the exclusive breastfeeding within the first 6 months of life on this association was then explored. We specifically tested for the association of antibiotic use in pregnancy with childhood asthma stratified by child gender and family history of allergic disorders and examined the modifiable effect of exclusive breastfeeding within the first 6 months of life on the association in high-risk children. Also examined were interactions between antibiotic use during pregnancy and (1) feeding patterns, (2) child sex, (3) family history and (4) child age, respectively, on the asthma development. Odds ratios (OR) and 95% confidence intervals (CIs) were calculated by multiple logistic regression models using SAS version 9.2 (IBM SAS Institute Inc., Cary, NC). The covariates adjusted in our models were: maternal age at delivery (years), maternal education level (≤9, 10–12, 13–16, ≥17 years, unknown), child age (years), child gender (female, male), gestational age at birth (weeks), delivery modes (cesarean delivery, virginal delivery), exclusive breastfeeding within the first 6 months of life (no, yes), family history of allergic disorders (no, yes, unknown). These covariates were selected according to prior knowledge [20, 21]. When performing adjustment, missing data of maternal education level and family history of allergic disorders were assigned to a category as ‘unknown’. When stratifying the analysis based on family history of allergic disorders, missing data were excluded.

## Results

Demographic characteristics were presented in Table 1. Compared to the mothers of the controls, the mothers of the cases were older and had a higher level of education. The asthma cases were younger, had more boys and a higher proportion of cesarean delivery, compared with the controls. The case group also had a higher prevalence of antibiotic exposure in pregnancy (7.1% versus 3.5%, *P* = 0.002). The prevalence of exclusive breastfeeding within the first 6 months of life, however, did not differ between the cases and controls (52.2% versus 53.8%, *P* = 0.5) (Table 1).

**Table 1:** Characteristics of the mothers and children and their associations with childhood asthma in the case - control study

Characteristics	Case (*n* = 634)	Control (*n* = 864)	*P*-value	OR	95% CI
Maternal educational level (years)			< 0.0001		
≤ 9	37 (5.8)	161 (18.6)		0.2	0.2–0.4
10–12	85 (13.4)	147 (17.0)		0.6	0.5–0.8
13–16	406 (64.0)	434 (40.2)		ref	ref
≥ 17	51 (8.0)	42 (4.7)		1.3	0.8–2.0
Unknown	55 (8.7)	80 (9.3)		0.7	0.5–1.1
Maternal antibiotic use in pregnancy*			0.002		
No	589 (92.9)	834 (96.5)		ref	ref
Yes	45 (7.1)	30 (3.5)		2.1	1.3–3.4
Maternal age at delivery (years)	28.5 (3.6)	27.7 (3.8)	0.0004	1.1	1.0–1.1
Gestational weeks at birth	38.8 (1.4)	38.9 (1.4)	0.1	0.9	0.9–1.0
Birth weight (g)	3305.6 (480.9)	3283.9 (500.4)	0.1	1.0	1.0–1.0
Delivery modes			0.02		
Vaginal delivery	237 (37.4)	376 (43.5)		ref	ref
Caesarean delivery	397 (62.6)	488 (56.5)		1.3	1.0–1.6
Exclusive breastfeeding within the first 6 months of life			0.5		
No	303 (47.8)	399 (46.2)		ref	ref
Yes	331 (52.2)	465 (53.8)		0.9	0.8–1.2
Child age (years)	5.7 (2.0)	6.3 (2.2)	< 0.0001	0.8	0.8–0.9
Child gender			0.002		
Female	252 (39.8)	414 (47.9)		ref	ref
Male	382 (60.2)	450 (52.1)		1.4	1.1–1.7
Child ethnicity			0.7		
Han	612 (96.5)	837 (96.8)		ref	ref
Other	16 (2.5)	22 (2.6)		1.0	0.5–1.9
Unknown	6 (1.0)	5 (0.6)		1.6	0.5–5.4
Family history of allergic disorders			< 0.0001		
No	289 (45.6)	679 (78.6)		ref	ref
Yes	334 (52.7)	173 (20.0)		4.5	3.6–5.7
Unknown	14 (1.7)	12 (1.4)		2.2	0.9–4.9

N (%) was used for maternal educational level, mother use of antibiotics in pregnancy, maternal age at delivery, delivery modes of children, exclusive breastfeeding within the first 6 months of life, child gender, child ethnicity, and family history of allergic disorders

Means (SD) was used for maternal age at delivery, gestational age at birth (weeks), birth weight, and child age

*Maternal use of antibiotics in pregnancy in our study excluded antibiotic use during labor or delivery

OR: odds ratios

Table 2 shows that prenatal use of antibiotics was significantly associated with an increased risk of childhood asthma after adjusting for potential confounders (adjusted OR [aOR]: 1.7, 95% CI: 1.0–2.9). When the subjects were stratified by feeding patterns, the association became non-significant among children with exclusive breastfeeding within the first six months of life (aOR 0.9, 95% CI 0.4–2.1), whereas the association became stronger in children without exclusive breastfeeding (aOR 2.6, 95% CI 1.3–5.1).

**Table 2:** Antibiotic use in pregnancy and the risks of childhood asthma stratified by feeding pattern within the first 6 months of life

Exposure categories	Case N (%)	Control N (%)	OR (95% CI)	aOR^a^ (95% CI)
Using antibiotics in pregnancy^b^ (*n* = 1498)				
No	589 (92.9)	834 (96.5)	ref	ref
Yes	45 (7.1)	30 (3.5)	2.1 (1.3–3.4)	1.7 (1.0–2.9)
Among children exclusively breastfed within the first 6 months of life (*n* = 796)				
No antibiotic use in pregnancy	318 (96.1)	452 (97.2)	ref	ref
Using antibiotics in pregnancy	13 (3.9)	13 (2.8)	1.4 (0.7–3.1)	0.9 (0.4–2.1)
Among children non-exclusively breastfed within the first 6 months of life (*n* = 702)				
No antibiotic use in pregnancy	271 (89.4)	382 (95.7)	ref	ref
Using antibiotics in pregnancy	32 (10.6)	17 (4.3)	2.7 (1.4–4.9)	2.6 (1.3–5.1)

^a^adjusted for maternal age at delivery (years), maternal education level (≤9, 10–12, 13–16, ≥17, unknown), child age (years), child gender (female, male), gestational age at birth (weeks), delivery modes (cesarean delivery, virginal delivery), family history of allergic disorders (no, yes, unknown)

^b^adjusted for exclusive breastfeeding within the first 6 months of life (no, yes) additionally

OR: odds ratios

aOR: adjusted odds ratios

Table 3 presents results stratified by gender, where boys with prenatal antibiotic exposure showed a higher risk than girls (for boys: aOR 2.2, 95% CI 1.1–4.4; for girls: aOR 1.3, 95% CI 0.5–3.0). However, the positive association between antibiotic use and asthma in boys was attenuated by exclusive breastfeeding (for boys with exclusive breastfeeding: aOR 1.0, 95% CI 0.4–3.1; for boys without exclusive breastfeeding: aOR 4.1, 95% CI 1.5–10.9).

**Table 3:** Antibiotic use in pregnancy and the risks of childhood asthma stratified by gender

Exposure categories	Case N (%)	Control N (%)	OR (95% CI)	aOR^a^ (95% CI)
Among boys^b^ (*n* = 832)				
No antibiotic use in pregnancy	349 (91.4)	436 (96.9)	ref	ref
Using antibiotics in pregnancy	33 (8.6)	14 (3.1)	2.9 (1.6–5.6)	2.2 (1.1–4.4)
Among girls^b^ (*n* = 666)				
No antibiotic use in pregnancy	240 (95.2)	398 (96.1)	ref	ref
Using antibiotics in pregnancy	12 (4.8)	16 (3.9)	1.2 (0.6–2.7)	1.3 (0.5–3.0)
Among boys				
Exclusively breastfed within the first 6 months of life (*n* = 437)				
No antibiotic use in pregnancy	185 (94.9)	234 (96.7)	ref	ref
Using antibiotics in pregnancy	10 (5.1)	8 (3.3)	1.6 (0.6–4.1)	1.0 (0.4–3.1)
Non - Exclusively breastfed within the first 6 months of life (*n* = 395)				
No antibiotic use in pregnancy	164 (87.7)	202 (97.1)	ref	ref
Using antibiotics in pregnancy	23 (12.3)	6 (2.9)	4.7 (1.9–11.9)	4.1 (1.5–10.9)

^a^adjusted for maternal age at delivery (years), maternal education level (≤9, 10–12, 13–16, ≥17, unknown), child age (years), gestational age at birth (weeks), delivery modes (cesarean delivery, virginal delivery), family history of allergic disorders (no, yes, unknown)

^b^adjusted for exclusive breastfeeding within the first 6 months of life (no, yes) additionally

OR: odds ratios

aOR: adjusted odds ratios

Table 4 further presents a stratified analysis by family history of allergic disorders. Children exposed to antibiotics in pregnancy and with a family history had a higher risk of asthma than those exposed to antibiotics in pregnancy but without family history (for children with family history: aOR 3.1, 95% CI 1.2–8.4; for children without family history: aOR 1.3, 95% CI 0.6–2.6). Exclusive breastfeeding also attenuated this positive association (for children with family history and exclusive breastfeeding: aOR 0.8, 95% CI 0.2–3.0; for children with family history and without exclusive breastfeeding: aOR 14.8, 95% CI 1.9–116.8).

**Table 4:** Antibiotic use in pregnancy and the risks of childhood asthma stratified by family history of allergic disorders

Exposure categories	Case N (%)	Control N (%)	OR (95% CI)	aOR^a^ (95% CI)
Among children with family history of allergic disorders^b^ (*n* = 507)				
No antibiotic use in pregnancy	303 (90.7)	168 (97.1)	ref	ref
Using antibiotics in pregnancy	31 (9.3)	5 (2.9)	3.4 (1.3–9.0)	3.1 (1.2–8.4)
Among children without family history of allergic disorders^b^ (*n* = 968)				
No antibiotic use in pregnancy	275 (95.2)	655 (96.5)	ref	ref
Using antibiotics in pregnancy	14 (4.8)	24 (3.5)	1.4 (0.7–2.7)	1.3 (0.6–2.6)
Among children with Family history of allergic disorders				
Exclusively breastfed within the first 6 months of life (*n* = 252)				
No antibiotic use in pregnancy	157 (94.6)	82 (95.4)	ref	ref
Using antibiotics in pregnancy	9 (5.4)	4 (4.6)	1.2 (0.4–3.9)	0.8 (0.2–3.0)
Non - Exclusively breastfed within the first 6 months of life (*n* = 255)				
No antibiotic use in pregnancy	146 (86.9)	86 (98.9)	ref	ref
Using antibiotics in pregnancy	22 (13.1)	1 (1.1)	13.0 (1.7–97.8)	14.8 (1.9–116.8)

^a^adjusted for maternal age at delivery (years), maternal education level (≤9, 10–12, 13–16, ≥17, unknown), child age (years), child gender (female, male), gestational age at birth (weeks), delivery modes (cesarean delivery, virginal delivery)

^b^adjusted for exclusive breastfeeding within the first 6 months of life (no, yes) additionally

OR: odds ratios

aOR: adjusted odds ratios

No significant interactions were found between antibiotic use during pregnancy and (1) feeding patterns, (2) child sex, (3) family history and (4) child age, respectively, on the asthma development (*P*-values were 0.06, 0.4, 0.1, 0.9, respectively).

## Discussion

Our hospital-based case-control study suggests that the prenatal exposure to antibiotics may increase the risk of asthma in children after controlling for confounders. This risk was particularly prominent in boys and children with a family history of allergic disorders. We also found that exclusive breastfeeding in the first six months of life may attenuate this risk particularly in children with these risk factors.

Our findings were consistent with most of previous studies on the association of prenatal antibiotic use with childhood asthma [4–8]. Over the past seventy-five years, antibiotic use surged from none to almost universal [22]. It has been reported that more than 40 % of pregnant women in the U.S. were prescribed pre-delivery antibiotics [9]. Although the immediate impacts of antibiotic administration in pregnancy are favorable [9], mouse and human studies suggested a potential long-term effect on the development of certain diseases in the offspring [23, 24].

Prenatal and extrauterine environmental factors are crucial to the development of immune system and the immune homeostasis. The mechanism underlying the association between prenatal antibiotics exposure and childhood asthma remains unclear. It was proposed that the antibiotic exposure may change the bacteria composition of maternal birth canal, intestinal tract and skin, which acts as primary nidi of newborn indigenous microbiota [9]. This antibiotic-related dysbiosis might persist and affect the maturation of infant’s nascent immunologic system and even lead to chronic immunologic disorders, including asthma [10, 11].

Our study found that exclusive breastfeeding in the first six months after birth may modify the association between prenatal antibiotics exposure and childhood asthma. Breastfeeding may provide appropriate intestinal milieu to increase the proliferation of health-promoting microorganisms [14]. A study reported that the infants fed by breast milk had more content of *Bifidobacteria* in intestine than those fed by infant formula [25]. Oligosaccharides have been identified as an important factor in breast milk that acts as metabolic substrate to stimulate the activation of *Bifidobacteria* [26]. Compared to the infants fed by infant formula, infants fed by breast milk also had a striking increase in the level of *Lactobacillus* species [25]. A mouse model [27] showed that *Lactobacillus salivarius* could improve the symptom of asthma through regulating the Th1/Th2 balance. The colonizing microbes contained in the breast milk may also originate from the skin of mother’s areola, infant mouth and even from maternal intestine [14, 28].

Animal studies showed that labeled bacteria placed in maternal gut could appear in breast milk. Although the number of maternal gut microbes that migrate into breast milk (10^3^/cc breast milk) is relatively small, these bacteria play a very important role in the intestinal colonization [11], which promotes immune development. For example, normal colonizing bacteria can ferment complex carbohydrates and produce short chain fatty acids, including butyrate. The latter can activate immunologic molecules and provide protection for immune function [11, 29]. Second, breast milk can promote the intestinal barrier to mature and secrete defensins, lysozyme, lactoferrin, polymeric IgA, soluble TLR-2 and 4, CD14 and MD2, which could invade pathogens [11, 30, 31]. Third, breast milk acts as a carrier transferring airborne antigens from mothers to neonates, induces immune tolerance and prevents children from allergic asthma [29, 32]. Our results suggest that exclusive breastfeeding may promote immune regulation and protect children from asthma, particular among those who were prenatally exposed to antibiotics.

Despite the consistent and innovative findings, this study has some limitations. First, we did not collect information on the reason for prenatal antibiotic use. Most antibiotics were prescribed to treat infection [33] . In utero fetal infection as one of the indications may affect the development of the fetal immune system. However, Stensballeet al. [4] found that the association between prenatal antibiotics exposure and childhood asthma remained robust in the group without a history of infection in pregnancy. Thus, the indication for antibiotic use may not be a substantial confounder. Second, we lack information on the dosage, type and duration of antibiotic use in pregnancy. McKeeveret al. [6] reported that the risks of childhood wheeze/asthma increased with the increased dosage of antibiotics use in pregnancy and presented a dose-response relationship. Metsala et al. [5] showed that the increased risk of childhood asthma was only associated with certain types of antibiotics in pregnant women. Lapin et al. [34] found that childhood asthma was significantly associated with antibiotic use in the second and third trimesters of pregnancy. But Mulder et al. [35] failed to show any difference in three trimesters.

Third, the prevalence of exclusive breastfeeding within the first six months after birth was 52.2% in the cases and 53.8% in the controls of this study, which appear higher than that reported in previous studies [36, 37]. The variation of sampling design, definition of exclusive breastfeeding and geographical location may to some degree explain the difference in prevalence. In addition, the national and local governments have made a large effort to promote exclusive breastfeeding in the first six months of life. A recent survey in Hunan Province of China showed that 44.9% of 1659 children younger than 5 years were reported to have been exclusively breastfed within the first 6 months after birth [38]. There was also a possibility that the mother might have misreported breastfeeding practice as breastfeeding occurred 3–12 years before the investigation. But previous studies demonstrated that the recall of breastfeeding was reliable even after 20 years [39]. Because there was a similar breastfeeding rate between the case and control groups in the present study, a differential recall bias was less likely. Unfortunately, as a retrospective study, we felt that it could be challenging to ask women to recall the exact duration of breastfeeding more than 3–12 years ago, and that a categorized duration of 6 months may be easier to remember. As mentioned above, the effort by national and local governments to promote exclusive breastfeeding in the first six months of life may facilitate the recall. Indeed, further prospective studies are needed to clarify the time effect of breastfeeding duration in the association between prenatal antibiotic use and childhood asthma.

Fourth, the participation rate of the control group was 78.6%, which was lower than that of the case group (91.0%). However, this difference was mainly due to our exclusion of controls who had history of either asthma or wheezing. If these exclusions are considered legitimate, then exclusions due to other reasons were similar between the cases and controls (Additional file 1). Finally, although we adjusted for a number of potential confounders, there may still be unmeasured confounders.

Our study has several advantages. First, the sample size was relatively large compared to many other studies; and the diagnosis of asthma was made by specialists. Second, our controls were selected from a wide range of pediatric departments. They better presented the source population of the cases. Additionally, inclusion of controls with different diseases may alleviate a bias if one disease was potentially related to the exposure. The generalizability of our study results needs further investigation.

## Conclusions

Our study suggests that antibiotic use in pregnancy was associated with an increased risk of childhood asthma, especially in boys and children with a family history of allergic disorders. Exclusive breastfeeding may attenuate this risk, especially among the high-risk children.

## Additional file

Additional file 1: **Table S1.** Characteristics of the mothers and children compared between participations and non-participations. **Table S2.** Characteristics of the mothers and children compared between participations and non-participations. If the variable had missing data, the comparation was examined between the mothers or children who had information. (DOCX 20 kb)

## Abbreviations

aOR: adjusted odds ratio
CIs: Confidence intervals
