# Supplementary material for: The effect of breastfeeding on the risk of asthma in high-risk children: a case-control study in Shanghai, China
Source: BMC Pregnancy Childbirth. 2018 Aug 23;18:341. doi: 10.1186/s12884-018-1936-5 (PMC6106762; doi:10.1186/s12884-018-1936-5)
Supplement: Supplementary file 1 — Table S1. Characteristics of the mothers and children compared between participations and non-participations. Table S2. Characteristics of the mothers and children compared between participations and non-participations. If the variable had missing data, the comparation was examined between the mothers or children who had information. (DOCX 20 kb) [file 12884_2018_1936_MOESM1_ESM.docx]

Supplementary table 1. Characteristics of the mothers and children compared between participations and non-participations.

| Characteristics | Participations  (n=1498) | Non-participations (n=298) | *P*-value |  |
| --- | --- | --- | --- | --- |
| **Maternal educational level (years)** | | | 0.7 |  |
| ≤9 | 198 (13.2) | 33 (11.1) |  |  |
| 10 - 12 | 232 (15.5) | 46 (15.4) |  |  |
| 13 - 16 | 840 (56.1) | 165 (55.4) |  |  |
| ≥17 | 93 (6.2) | 21 (7.1) |  |  |
| Unknown | 135 (9.0) | 33 (11.1) |  |  |
| **Maternal antibiotic use in pregnancy^*^** | |  | <0.0001 |  |
| No | 1423 (94.5) | 191 (86.0) |  |  |
| Yes | 75 (5.0) | 10 (4.5) |  |  |
| Unknown | 0 (0.0) | 21 (9.5) |  |  |
| **Maternal age at delivery (years)** | 28.1 (3.7) | 28.2 (3.8) | 0.5 |  |
| **Gestational weeks at birth** | 38.9 (1.3) | 38.5 (1.8) | <0.0001 |  |
| **Birth weight (g)** | 3312.2 (469.2) | 3189.0 (571.2) | 0.0005 |  |
| **Delivery modes** |  |  | <0.0001 |  |
| Vaginal delivery | 885 (59.1) | 197 (66.1) |  |  |
| Caesarean delivery | 613 (40.1) | 85 (28.5) |  |  |
| Unknown | 0 (0.0) | 16 (5.4) |  |  |
| **Exclusive breastfeeding within the first 6 months of life** | |  | <0.0001 |  |
| No | 702 (46.9) | 169 (56.7) |  |  |
| Yes | 796 (53.1) | 122 (40.9) |  |  |
| Unknown | 0 (0.0) | 7 (2.4) |  |  |
| **Child age (years)** | 6.0 (2.1) | 5.7 (2.4) | 0.08 |  |
| **Child gender** |  |  | 0.5 |  |
| Female | 666 (44.5) | 126 (42.3) |  |  |
| Male | 832 (55.5) | 172 (57.7) |  |  |
| **Child ethnicity** | | | 0.5 |  |
| Han | 1449 (96.7) | 285 (95.6) |  |  |
| Other | 38 (2.5) | 11 (3.7) |  |  |
| Unknown | 11 (0.7) | 2 (0.7) |  |  |
| **Family history of allergic disorders** | | | <0.0001 |  |
| No | 968 (64.6) | 188 (63.1) |  |  |
| Yes | 507 (33.8) | 88 (29.5) |  |  |
| Unknown | 23 (1.5) | 22 (7.4) |  |  |

N (%) was used for maternal educational level, mother use of antibiotics in pregnancy, maternal age at delivery, delivery modes of children, exclusive breastfeeding within the first 6 months of life, child gender, child ethnicity, and family history of allergic disorders.

Means (SD) was used for maternal age at delivery, gestational age at birth (weeks), birth weight, and child age.

Maternal use of antibiotics in pregnancy in our study excluded antibiotic use during labor or delivery.

Supplementary table 2. Characteristics of the mothers and children compared between participations and non-participations. If the variable had missing data, the comparation was examined between the mothers or children who had information.

| Characteristics | Participations | Non-participations | *P*-value |  |
| --- | --- | --- | --- | --- |
| **Maternal educational level (years)** | | | 0.9 |  |
| ≤9 | 198 (13.2) | 33 (11.1) |  |  |
| 10 - 12 | 232 (15.5) | 46 (15.4) |  |  |
| 13 - 16 | 840 (56.1) | 165 (55.4) |  |  |
| ≥17 | 93 (6.2) | 21 (7.1) |  |  |
| **Maternal antibiotic use in pregnancy^*^** | |  | 1.0 |  |
| No | 1423 (95.0) | 191 (95.0) |  |  |
| Yes | 75 (5.0) | 10 (5.0) |  |  |
| **Maternal age at delivery (years)** | 28.1 (3.7) | 28.2 (3.8) | 0.5 |  |
| **Gestational weeks at birth** | 38.9 (1.3) | 38.5 (1.8) | <0.0001 |  |
| **Birth weight (g)** | 3312.2 (469.2) | 3189.0 (571.2) | 0.0005 |  |
| **Delivery modes** |  |  | 0.0007 |  |
| Vaginal delivery | 885 (59.1) | 197 (69.9) |  |  |
| Caesarean delivery | 613 (40.1) | 85 (30.1) |  |  |
| **Exclusive breastfeeding within the first 6 months of life** | |  | 0.0005 |  |
| No | 702 (46.9) | 169 (58.1) |  |  |
| Yes | 796 (53.1) | 122 (41.9) |  |  |
| **Child age (years)** | 6.0 (2.1) | 5.7 (2.4) | 0.08 |  |
| **Child gender** |  |  | 0.5 |  |
| Female | 666 (44.5) | 126 (42.3) |  |  |
| Male | 832 (55.5) | 172 (57.7) |  |  |
| **Child ethnicity** | | | 0.3 |  |
| Han | 1449 (97.4) | 285 (96.3) |  |  |
| Other | 38 (2.6) | 11 (3.7) |  |  |
| **Family history of allergic disorders** | | | 0.4 |  |
| No | 968 (65.6) | 188 (68.1) |  |  |
| Yes | 507 (34.4) | 88 (31.9) |  |  |

N (%) was used for maternal educational level, mother use of antibiotics in pregnancy, maternal age at delivery, delivery modes of children, exclusive breastfeeding within the first 6 months of life, child gender, child ethnicity, and family history of allergic disorders.

Means (SD) was used for maternal age at delivery, gestational age at birth (weeks), birth weight, and child age.

Maternal use of antibiotics in pregnancy in our study excluded antibiotic use during labor or delivery.

Participations and non-participations with missing data of maternal antibiotic use in pregnancy (n=21), delivery modes (n=16), child ethnicity (n=13), exclusive breastfeeding within the first 6 months of life (n=7), family history of allergic disorders (n=45) were excluded and compared, respectively.
